# Global collaboration and social practices to mitigate impacts of COVID-19 in the world: a lived experience of infecting

**DOI:** 10.1177/1473325020981088

**Published:** 2021-03

**Authors:** Mozhgan Moshtagh, Jila Mirlashari, Rana Amiri

**Affiliations:** Social Determinants of Health Research Center, Faculty of Health, Birjand University of Medical Sciences, Birjand, Iran; Department of OBGYN, Women’s Health Research Institute, University of British Columbia, Vancouver, Canada; Tehran University of Medical Sciences, School of Nursing and Midwifery, Tehran, Iran; Department of Geography and Environmental Sciences, Northumbria University, Newcastle, UK; Post graduate psychology provision department, University of Sunderland, Sunderland, UK

**Keywords:** COVID-19 pandemic, consequences, global collaboration, social work and practice, reflexive essay

## Abstract

COVID-19 pandemic is one of the most serious threatening conditions and the complex situation in the recent century, which shook the world. This unprecedented crisis has caused many disruptions and distractions for humans in different local and global levels. This reflexive essay aims to review challenges and opportunities originated by the Corona-virus pandemic within social groups through a moral perspective. Focusing on both negative and positive aspects would help us find the required skills and strategies to adapt to the crises and mitigate the issues based on our capacities and resources.

## Introduction

Our planet has been encountering with numerous uncontrollable disasters in the recent century that Covid-19 is one of them. It could be considered a bio-psycho-social crisis due to severe damage created in the global health and the economy. Humankind, regardless of ethnicity, region, social class, gender, and age, faces with an unpredictable and life-threatening situation. Millions of people are struggling with its subsequent complications, while thousands of them are failed against this fatal virus. Financial bankrupting in different industries and organizations have raised many economic and psychosocial problems. The shortage of drugs, medical equipment and protective supplies, and widespread overcrowding in the hospitals created an arduous situation for care providers and governments.

Fear of a deadly and unknown disease, the uncertainty of curbing and controlling the virus, lockdown and sense of isolation and other subsequent consequences such as disability, food insecurity, and financial shortage are some of Coronavirus’ burdens. Furthermore, perceived stigma, feeling rejected, and helplessness among infected individuals and their families have a drastic impact on their mental health (Bowles et al., 2016; Li et al., 2020; Shigemura et al., 2020; Wang et al., 2020).

Collapse in the essential sectors such as trading and business may have long-term or irreversible consequences. Quarantine and social isolation have changed people’s lifestyles, disrupted personal and social living routines, and increased family distress (Brooks et al., 2020). This article aims to illuminate some negative and positive impacts of COVID-19 as a uniquely personal experience.

### COVID-19 as a unique experience

COVID-19 outbreak is an exceptional experience, which we will never forget throughout our life. Although everyone has experienced some negative emotions during COVID-19, we should not ignore the positive effects. While it has created various problems for many people around the world, it is essential to learn about the impacts and find some strategies to alleviate the adverse effects.

My previous lived experiences as a nurse and my present experience as a researcher, who has infected with Coronavirus, opened my eyes to focus on the impacts of COVID-19 crisis in developing countries and vulnerable populations. Around February 8, 2020, I had to take several travels during the Coronavirus outbreak to provide care for my mother with a vertebra fracture. This travels took place in a situation that protective equipment and disinfectants were scarce in Iran. After a week, I felt malaise and had a sore throat but did not consider it seriously, believing that it might be a cold. I went to the hospital when signs of fever, chill, and dyspnea appeared. I was shocked and confused when the para-clinical tests verified that I was infected with COVID-19. I was suggested for self-isolation while I was worried about my disabled and old mother, which I could no longer take care of her.

### Negative effects of COVID-19

#### Stigma

Facing a viral outbreak can be associated with many negative feelings including anxiety about judgment and individuals’ reactions in society (Wang et al., 2020). Waiting for getting the test results, I was at the emergency department that a young lady captured my attention. She, who was a family member of a patient near my bed, had terribly scared of me and asked a nurse about my medical diagnosis. I felt that other people, even health care providers, were afraid of me and tried to stay away from me. As someone who has had nursing experience for years, I felt be ashamed and embarrassed because it was such an inexperienced challenging moment in my life.

#### Isolated and alone

Although many people have experienced being lonely during quarantine, the situation was much harder for individuals who were infected and became sick. Besides, COVID-19 outbreak happened simultaneously with the advent of the Iranian New year, which is one of our most important events. As a family, we had to stay away from each other and start New Year alone by ourselves, which never had been experienced throughout our life. I was not feeling well at all and had to fight with an unknown and dangerous virus. Fear of death, accompanied by feelings of loneliness and anxiety, pervaded my all time. I spent many days alone and in fear worried about my children and family. Virtual contacts with family, relatives and friends were the only rays of hope to resist and became resilient.

#### Women issues

Women who have a caring role in the family are susceptible to experience emotional wreck and mental problems (Saghazadeh and Rezaei, 2020; Shigemura et al., 2020). Although evidence indicates a higher mortality rate among men, it is clear that women are more vulnerable to psychological consequences such as post-traumatic stress, anxiety, and depression (Lim et al., 2018; Rubin et al., 2010; Zhang and Ma, 2020). As a woman I experienced various stress and anxious when I was infected with Covid-19. I kept separated from my children and my family for 3 weeks, just seeing them by consoles. It was a tough condition because while I was full of pain, worried about my children and my mother made me more fragile. I thought that who could care for my poor mum, which was at significant risk of catching the disease and could not do her routine activities. I had badly missed my children and thought about how they could care for themselves and what happens if they were infected. All those issues made me so wreck and weak that I was crying without any reasons and became over-sensitive even after recovery.

#### Inter family challenges

Also, staying at home, changing lifestyle, and restricting social engagement has surged psychosocial burdens within families. Parental stress has increased due to the coincidence of different roles such as remote working, doing chores, assisting children for their homework, and providing safe and happy moments for children to enjoy. Such condition would exacerbate the stressful situation for impoverished families who are living in small and cramped households and may raise the incidence of violence against children or child abuse (Bradbury‐Jones and Isham, 2020; Child, 2020; Moshtagh et al., 2017, 2018). Violence against women has also increased, according to some reports (Bradbury‐Jones and Isham, 2020).

Besides, children and adolescents might be at risk of low physical activity and too much sedentary time, excessive screen time or gaming, and consuming snacks, which jeopardize their physical and mental well-being. Older children and adolescents may experience more challenges to deal with social distancing and isolation. They are in a critical developmental stage to reach independence; therefore, they need to contact with their peers and friends. Moreover, they might not be resilient enough to adapt to a lack of predictability and security about the future of education, life, family, and friends.

#### Scarce medical equipment and social health support

If I had access to protective equipment such as gloves and masks, I might never be sick and would never experience that terrible journey. When Corona-virus was started to spread in different Iran regions, it was hard to find masks and sanitizers. Everything, especially food and fruits became too expensive, and there were lots of health equipment shortages even in the hospitals for health personnel. Increasing the amount of fraudulent and uncontrolled business for masks and sanitizers made the situation much worse for the citizens. During my illness period, I desperately required someone to give me some psychological counseling. Still there was no one to protect ill people, and they should have just stayed at home without any attention or follow-ups. Also, there are many hardships and barriers to access and use health services and social facilities, especially for marginal or deprived populations.

#### Community problems

Elderlies and individuals with chronic disease or disability (physical or psychological) are the most vulnerable groups faced with significant physical and psychosocial threats. According to the evidence, most of the death cases due to COVID-19 have been old or had underlying diseases (Cesari and Proietti, 2020; Chow et al., 2020). Therefore, fear of getting severe infection and death is much higher among them. Moreover, lockdown and isolation have restricted their social contribution and networks, which can induce feelings of loneliness, depression, and distress (Pérez et al., 2019).

People with chronic conditions, pregnant women, and infants may not have access to proper services and care (Hayden, 2015; Menéndez et al., 2015; Takahashi et al., 2015; Walker et al., 2015). On the other hand, mental ills and persons with addiction would have more difficulty coping with quarantine and probably tend to self-harm or demonstrate aggressive behaviors and are also at risk for fatal overdose and death (Tattevin et al., 2015; Volkow, 2020). Generally, threatening events, including loss of beloved families and friends and perceived personal crisis during the COVID-19 outbreak was associated with a sense of existential loneliness, meaninglessness, and hopelessness (Ettema et al., 2010).

### Positive effects of COVID-19

#### We need people to have a happy life

Being isolated at home and quarantine proved that people around us play an important role in our mental health. While in the past I preferred to be alone and far from of population, now I recognized the importance of crowd in my life. When I went to the Town Centre for the first time during quarantine, and I observe it completely avoid people, I cried, scared and my heart was broken. My mother could not walk or go outside and when she missed people, she was sitting behind the window and watched people.

I recognized the friends who care about me in difficult conditions, and I learned to be grateful for all the moments I have. I can enjoy more for hugging, kissing and touching each other, which we thought they are routine, and Corona broke this sweet everyday actions.

#### Family takes more meaning and they spend more time with each other

During Corona virus pandemic many people stayed at home. The parents who were always busy and had not enough time to spend with children joined to the family. They went outside to do exercise, cycling and perceived the meaning of a family and parenthood. They ate breakfast, lunch, and dinner as a family and they had more time to speak with each other without tension and pressure. They hugged and kissed more than before, told more night stories to their children, and provided their favorite dishes without stress or fear of becoming late.

#### My perspective on life changed

During infecting with Corona-virus I experienced a roller coaster of emotions. First, I was terribly scared of dying and worried sick about my children and my mother. I was sleeping with excruciating pain and chilling fever and opening my eyes while I thought I might died. After a while, my symptom became milder, but emotionally I was completely wrecked and deeply depressed. My perspective on life has changed greatly, and I found the meaning of beautiful life. These days when I am opening my eyes in the morning after a nice sleep, I feel blissfully happy and pleasantly thankful to have a healthy and happy life.

Having a breath without difficulty, sleeping without pain and fever and having an appetite for eating food and recognizing its taste taught me to be pleased and enjoy every minute of life.

#### Seek help, hope, and meaning as a coping strategy

Despite many adversities caused in personal and community living owing to COVID-19 influences, a positive trend has been shaped in some societies. These situations have induced people to provide social support and care for each other, especially for vulnerable groups. They have been trying to find hope and meaning by connecting and sharing their experiences and information via social media. Individuals have been making companions with people who have experienced worse conditions in their community or the world to overcome frightening situations. Religious people have been engaging in rituals for praying for hope and peace, and others have been using spiritual and non-spiritual ways to share support and security (Kim, 2018; Lane et al., 2019; Mak et al., 2009).

I was grateful for receiving supports via social media and was thankful that this modern technology helped me be strong during this hard time. I found many videos shared by families and friends and academic individuals on WhatsApp and Instagram, and I owe all of them forever. I shared all the vidoes after my recovery as it seems beneficial for people who have the same situation. 

#### Local and global collaboration to mitigate the crisis

Celebrities and wealthy people contributed to fundraising for deprived and demanding groups. Volunteer members of different ages and classes have been collaborating by packing the necessary items for needy people and distributing protective equipment in poor communities or disinfected streets and the city areas. Some community groups developed to deliver services and non-financial aids to beneficiaries. Public education and raising awareness are held via campaigns, virtual networks, media, and telemedicine to help people for prevention, screening, and even recovering.

Personal protective equipment and testing kits are provided for many countries by WHO. National and international plans are made to manage this borderless crisis. Global scientists invited to brought together to discover medicinal substances or vaccines to cure the disease. Because of the extremely detrimental impacts of the crisis, all societies are accountable for controlling the virus. According to international health regulations, countries have been committing to prevent the spread of the virus and support less developed societies in order to fundability for dealing with the risks (Board, 2019). Social workers can help people by empowering them to identify crucial needs or demands in vulnerable groups and increase knowledge, hope, and spirituality within communities.

This situation is a golden opportunity to practice altruism. I decided to provide masks and sanitizers for people who cannot afford, called to elderly and disabled people who were in long quarantine to have a nice chat, and do the shopping for neighbors who were ill or old. Many other people in our city did these kinds of philanthropic actions. It was not a big job or spending big money, but we tried to do whatever we could to make the situation a little better.

### Lessons

Despite the drawbacks and drastic impacts created on the world, the Coronavirus has had some positive influences on individuals and societies. Although quarantine has made bodies distant, it has closed hearts to each other. Altruistic values, social responsibility, parental responsibility, and spiritual desires have risen among people. It proved that economic development is associated with social and human interactions. We learned that sustainable development would require maintaining global equality and equity in health and welfare.

## Conclusion

Experiencing COVID 19 may be a cumbersome event, but it taught us lifelong lessons. It opened our eyes to invaluable and invariable values such as humanity and compassion. These feelings give us the trust and keep our safety when facing uncertainty and high-risk situations. We understood that morality and spirituality is pre-requisite for sustainable development and global well-being.

The world is like a village or a united body that problems in every part could transmit to other ones. Philanthropic individuals, care providers, and social workers are like an immune-cell that protects other lives. They help people to defend against disease, suffering, and adversity without considering race, ethnicity, or religion even though, a long-lasting fight might lead to harmful impacts on their health. Thus, global equity, peace, health, and welfare are essential materials that would enhance social soldiers’ well-being or helper identities.
